# Network Analysis for the Discovery of Common Oncogenic Biomarkers in Liver Cancer Experimental Models

**DOI:** 10.3390/biomedicines11020342

**Published:** 2023-01-25

**Authors:** Loraine Kay D. Cabral, Pablo J. Giraudi, Gianluigi Giannelli, Francesco Dituri, Roberto Negro, Claudio Tiribelli, Caecilia H. C. Sukowati

**Affiliations:** 1 Fondazione Italiana Fegato ONLUS, AREA Science Park, Campus Basovizza, 34149 Trieste, Italy; kay.cabral@fegato.it (L.K.D.C.); pablo.giraudi@fegato.it (P.J.G.); ctliver@fegato.it (C.T.); 2 Doctoral School in Molecular Biomedicine, University of Trieste, 34127 Trieste, Italy; 3 National Institute of Gastroenterology IRCCS “S. De Bellis” Research Hospital, 70013 Bari, Italy; gianluigi.giannelli@irccsdebellis.it (G.G.); francesco.dituri@irccsdebellis.it (F.D.); roberto.negro@irccsdebellis.it (R.N.); 4 Eijkman Research Center for Molecular Biology, National Research and Innovation Agency of Indonesia (BRIN), Jakarta Pusat 10340, Indonesia

**Keywords:** hepatocellular carcinoma, cellular heterogeneity, targeted therapies, experimental models

## Abstract

Hepatocellular carcinoma (HCC) is a malignancy marked by heterogeneity. This study aimed to discover target molecules for potential therapeutic efficacy that may encompass HCC heterogeneity. In silico analysis using published datasets identified 16 proto-oncogenes as potential pharmacological targets. We used an immortalized hepatocyte (IHH) and five HCC cell lines under two subtypes: S1/TGFβ-Wnt-activated (HLE, HLF, and JHH6) and the S2/progenitor subtype (HepG2 and Huh7). Three treatment modalities, 5 µM 5-Azacytidine, 50 µM Sorafenib, and 20 nM PD-L1 gene silencing, were evaluated in vitro. The effect of treatments on the proto-oncogene targets was assessed by gene expression and Western blot analysis. Our results showed that 10/16 targets were upregulated in HCC cells, where cells belonging to the S2/progenitor subtype had more upregulated targets compared to the S1/TGFβ-Wnt-activated subtype (81% vs. 62%, respectively). Among the targets, FGR was consistently down-regulated in the cell lines following the three different treatments. Sorafenib was effective to down-regulate targets in S2/progenitor subtype while PD-L1 silencing was able to decrease targets in all HCC subtypes, suggesting that this treatment strategy may comprise cellular heterogeneity. This study strengthens the relevance of liver cancer cellular heterogeneity in response to cancer therapies.

## 1. Introduction

The global burden of hepatocellular carcinoma (HCC) contributed to around 900,000 new cases worldwide in 2020. It ranked as the sixth most common malignancy and third most common cancer-related death worldwide [1]. Different etiologies such as chronic viral hepatitis B and C, alcohol abuse, metabolic syndromes, and aflatoxin exposure have been attributed to causing HCC [2]. This type of malignancy has been described to be molecularly complex primarily because of the heterogeneity of the tumors [3]. This heterogeneity contributes largely to the chemoresistant nature of HCC [4]. About two-thirds of HCC cases are diagnosed in the advanced and metastatic stages [5]. Unfortunately, only a handful of therapies can offer significant treatment effects to HCC patients in the late-advanced stages.

To date, several tyrosine-kinase inhibitors (TKIs) are recommended for the first-line treatment of advanced HCC, namely, Sorafenib [6] (approved in 2007) and Lenvatinib [7] (approved in 2018). With the recent development of immunotherapy for cancer, HCC has benefited from immune checkpoint inhibitors (ICIs) among patients with or without prior Sorafenib treatment. Nivolumab [8] and Pembrolizumab [9], antibodies against the programmed cell death protein (PD-1) are already approved as second-line treatments for HCC [10]. Combinations between Atezolizumab (anti-PD-L1, a PD-1 ligand) with Cabonzantinib, an anti-vascular endothelial growth factor receptor (VEGFR), showed higher efficacy [11] prompted their use as first line treatments in advanced HCC [12]. Moreover, ICIs against PD-L1 used on treatment-naïve patients with unresectable HCC showed an acceptable low side-effect profile and promising antitumor activity [13]. However, despite these improvements in HCC therapy, there are only 15% of HCC eligible for potentially curative treatments [14], which dictates the need to discover other pharmacological or molecular targets that will provide a better therapeutic potential. 

Several clinical and histopathologic evidence describes HCC as a heterogeneous disease, but there is still a need to provide a coherent molecular explanation for HCC heterogeneity [15]. Several researchers have utilized -omics approaches to classify HCC, focusing on their molecular and cellular taxonomies [15,16,17]. These classifications resulted in the so-called molecular classes/subtypes that reflect the heterogeneity of the cells. Each class/group/subtype shows distinct cellular phenotypes, (dis)activations of molecular pathways, differentiation, and sensitivities to given treatments. This study, therefore, aimed to look at potential targets for HCC treatment, taking advantage of these reported molecular classifications together with bioinformatics tools. The exploration of the validity of proposed targets for the treatment of HCC was assessed in experimental models comprising different cellular classifications. 

## 2. Materials and Methods

### 2.1. Selection of Targets

**Figure 1:**
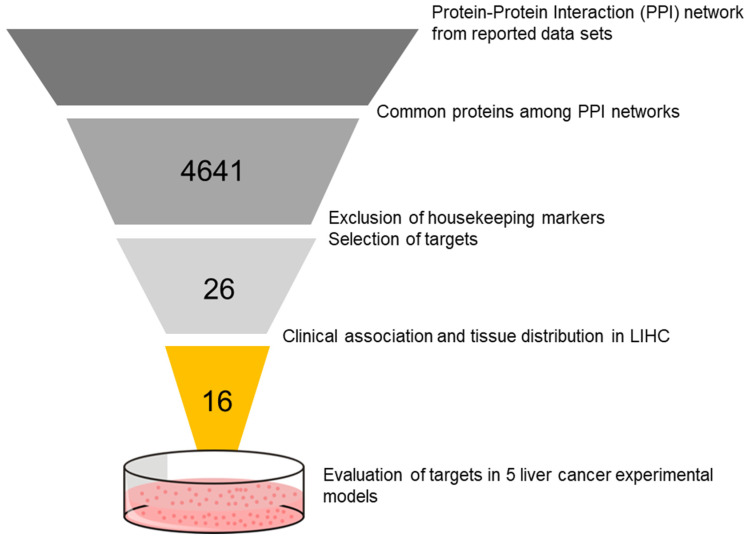
Simplified graphical illustration of the in silico and validation approach to discover potential targets for HCC therapy.

We looked into published datasets of HCC transcriptomic profiles as presented by Boyault et al., and Hoshida et al. [15,16]. The two publications proposed groups and subtypes for HCC based on the similarity of cellular and molecular signatures of tumors. Using these datasets, a protein–protein interaction (PPI) analysis was done using Cytoscape [18] to select common proteins from the PPI network. The gradual screening to select candidate targets was done by excluding housekeeping genes and focusing on genes that were involved in cancer promotion (proto-oncogenes). The clinical association and significance of each proto-oncogene to LIHC (liver hepatocellular carcinoma) was plotted into data from The Cancer Genome Atlas (TCGA) and the Genotype Tissue Expression (GTEx) portals [19,20], and visualized by the Gene Expression Profiling Interactive Analysis (GEPIA) online tool [21]. Figure 1 shows a diagram of the in silico strategy used in this study, while the generated PPI networks from datasets are shown in Figure S1 of Supplemental Data. 

### 2.2. Classification and Characterization of In Vitro Models

We selected representative cell lines that correspond to the different subtypes of HCC tumors. Six cell lines, which consisted of 1 immortalized hepatocyte and 5 HCC cell lines, were used for in vitro analysis. The HCC cell lines HLE, HLF, and JHH6 were classified under the subtype 1/transforming growth factor beta–Wingless related integration site (S1/TGFβ-Wnt) activated subtype, and HepG2 and Huh7 were classified as subtype 2 (S2/progenitor subtype) [3]. All cell lines were grown in their respective culture media supplemented with 10% (*v*/*v*) fetal bovine serum (FBS), 1% L-glutamine, and 1% antibiotics. Dulbecco’s Modified Eagle’s Medium (DMEM)-F12 medium was used for the immortalized hepatocytes IHH with additional supplements of 1 µM dexamethasone, and 5 µg/mL insulin. DMEM medium (high glucose) was used for HCC cells, except for JHH6 which was cultured in Williams’ E medium. Cells were maintained at 37 °C in a humidified 5% CO_2_ incubator. Routine cell expansion was performed using 0.05% trypsin detachment when cells achieved 80% cell confluency. Flow cytometry analysis was used to characterize the cell populations according to groups and subtypes that have been reported in our previous study [22].

### 2.3. Evaluation of Targeted Therapies in In Vitro Models

For in vitro treatment, three targeted therapies were conducted, consisting of the following: 5-Azacytidine (5-AZA), an epigenetic therapy acting as a DNA methyltransferase (DNMT) inhibitor; Sorafenib (SOR), a tyrosine kinase inhibitor; and PD-L1 mRNA silencing by small interference RNA (siR-PD-L1). 

Each cell line was seeded at 25,000 cells/cm^2^, except for JHH6 at 12,500 cells/cm^2^. Cytotoxicity experiments were performed to define the lethal concentration (LC_50_) of 5-AZA and SOR. For the evaluation of 5-AZA (A2385, Sigma-Aldrich, St. Louis, MO, USA), each cell line was treated with concentrations ranging from 2 µM to 5 mM, as reported in our previous work defining the non-toxic concentration of 5-AZA [22], while for the evaluation of SOR (Nexavar^®^, Bayer, Leverkusen, Germany), the cell lines were exposed to concentrations from 1 to 80 uM. Cell viability was evaluated after 24 h of drug exposure using the 3(4,5-dimethyl thiazolyl-2)-2,5 diphenyltetrazolium assay (MTT, Sigma Aldrich) to determine the LC_50_ of the drug to each cell line. 

Gene-silencing experiments for PD-L1 were performed using 20 nM of siRNA PD-L1 (Hs siRNA against CD274 (Thermo Fisher, Waltham, MA, USA). The siRNAs were transfected into cells using siLentFect^TM^ Lipid Reagent (170–3362, Bio-Rad, Hercules, CA, USA) according to the manufacturer’s instructions. Control siRNA (sc-37007, Santa Cruz Biotech, Dallas, TX, USA) was included in each assay. Cells were exposed to siRNA for 48 h, followed by cell collection for RNA and total protein extraction prior to PD-L1 gene expression and Western blot analysis.

### 2.4. Collection of Total RNA and Protein from Treated Cells

Following all treatments, all cells were washed twice with cold phosphate-buffered saline (PBS) solution and then suspended in at least 500 µL of Tri Reagent^®^ (Sigma-Aldrich) for RNA and protein extraction according to the manufacturer’s instruction. RNA was quantified at wavelength 260 nm in a spectrophotometer (Beckman Coulter, Brea, CA, USA) and RNA purity was evaluated according the Minimum Information for Publication of Quantitative Real-Time PCR Experiments (MIQE) guidelines by measuring the ratio A260/A280 with an appropriate purity value between 1.8 and 2.0 [23]. The integrity of RNA was assessed on standard 1% agarose/formaldehyde gel. Protein concentration was determined by the bicinchoninic acid protein assay (BCA). At least three replicates were evaluated for each cell lines and for each treatments.

### 2.5. Quantitative Real-Time PCR (RT-qPCR)

One microgram of purified RNA was subjected to cDNA synthesis using the High Capacity cDNA Reverse Transcription Kits (Applied Biosystems), according to the manufacturer’s protocol. Real-time PCR was performed in CFX 9600 real-time PCR system (Bio-Rad) according to the PowerUp SYBR Green mix protocol (Applied Biosystems, Waltham, MA, USA). Briefly, gene amplification was carried out in a 15 µL PCR reaction volume containing 1X PowerUp SYBR Green mix, 250 nM of gene-specific forward and reverse primers and 25 ng cDNA. The primer sequences to analyze the 16 gene targets and the housekeeping β-actin gene used in this study are listed in Table 1. 

### 2.6. Western Blot Analysis

Protein expressions from treated cells were evaluated using Western blot (WB) analysis. A total of 10 µg of protein lysates was loaded onto 10% polyacrylamide sodium dodecyl–sulfate polyacrylamide gel electrophoresis (SDS-PAGE) and then wet-transferred onto a polyvinylidene difluoride (PVDF) membrane. Following blocking, membranes were washed and incubated with primary antibodies against c-Src (recognizing c-Fgr) (sc-8056, Santa Cruz Biotech) for 24 h. Anti-actin (A2066, Sigma-Aldrich) was used as a housekeeping protein. Secondary antibodies were anti-mouse IgG HRP (Dako-p0260) and anti-rabbit IgG HRP (Dako-p0448), depending on the first antibody. Membranes were washed and then exposed to ECL Plus WB detection system solutions (ECL Plus Western Blotting Detection Reagents, GE-Healthcare Bio-Sciences) to obtain peroxidase reaction. The blots were visualized using a C-Digit blot scanner and analyzed using Image Studio™ Vers. 5.2 Acquisition software (LI-COR Biosciences). Protein relative quantification was performed after the densitometric analysis of bands vs. actin in each sample.

### 2.7. Statistical Analysis

Statistical significance was calculated using software GraphPad Prism version 8.0 (GraphPad Software, San Diego, CA, USA) mRNA and protein expression data are presented as mean ± SD/SEM. The difference between groups was calculated using the *t*-test. To determine statistical significance, the *p*-value was set to 0.05 and reported as * *p* < 0.05, ** *p* < 0.01, and *** *p* < 0.001.

## 3. Results

### 3.1. Identification of Candidate Targets

We employed an in silico strategy to consider the innate heterogeneity of HCC by gradual filtering, to discover potential drug targets that may comprise cellular heterogeneity. From the PPI networks (Figure S1), we identified 982 and 3659 common proteins from Hoshida and Boyault extended classifications, respectively. Gradual selection from those proteins, by excluding housekeeping genes and including proto-oncogenes, resulted in 26 proto-oncogene targets. From those targets, following GEPIA analysis on their clinical distributions and associations according to TCGA and GTEx datasets (comprising 369 liver cancer tissues vs. 160 normal tissues), we further narrowed down the targets to 16 candidates as shown in Table 2. 

### 3.2. Expression of Targets in the Cell Populations

**Figure 2:**
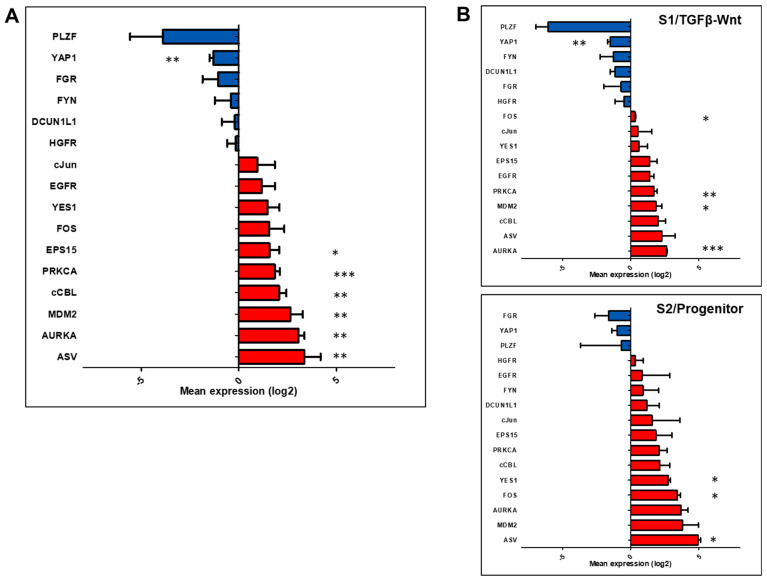
Baseline mRNA expression of targets in the various HCC cell populations. (**A**) Distribution of targets showing the upregulated and the down-regulated proto-oncogenes. (**B**) Distribution of relative expression of candidate targets between the S1 and S2 cell populations. Data are presented as the mean expression values (log2) from three independent samples of each cell lines. Statistical analysis: * *p* < 0.05, ** *p* < 0.01, *** *p* < 0.001 using the one-sample *t*-test vs. mean expression of immortalized hepatocytes IHH as 0.00. Red—up-regulated, Blue—down-regulated.

We then analyzed the baseline expression levels of the 16 targets (Table 2) in the in vitro models, comparing their expressions in HCC cells to those in IHH. We observed that 10 out of the 16 (62%) genes were up-regulated and 6 were down-regulated in the HCC cell lines (Figure 2A). Further comparison of gene expressions between the two HCC cell classifications showed that 13 out of the 16 proto-oncogene targets (81%) were up-regulated in the S2/progenitor subtype, as compared to 10/16 (62%) in the S1/TGFβ-Wnt subtype (Figure 2B). 

### 3.3. Effect of Targeted Treatments on Different Cell Populations

Previously, we evaluated the experimental models used in this study using cancer stem cells (CSC) markers to confirm the heterogeneity of the cells [22]. Results showed the presence of epithelial cell adhesion molecule (EpCAM)-positive cells on the S2/progenitor subtype cells (HepG2 and Huh7).

We used three different treatment strategies in the different HCC cell populations. In our previous study, LC_50_ of 128 μM for HLE, 33 μM for HLF, 41 μM for IHH, 16 μM for Huh7, 14 μM for HepG2, and 5 μM for JHH6 were determined. We chose the concentration of 5 µM as a non-lethal concentration for 5-AZA epigenetic therapy. This concentration was able to inhibit the methylation activities of DNMT1 allowing the reversal of transcriptional silencing, as seen in our previous data [22].

**Figure 3:**
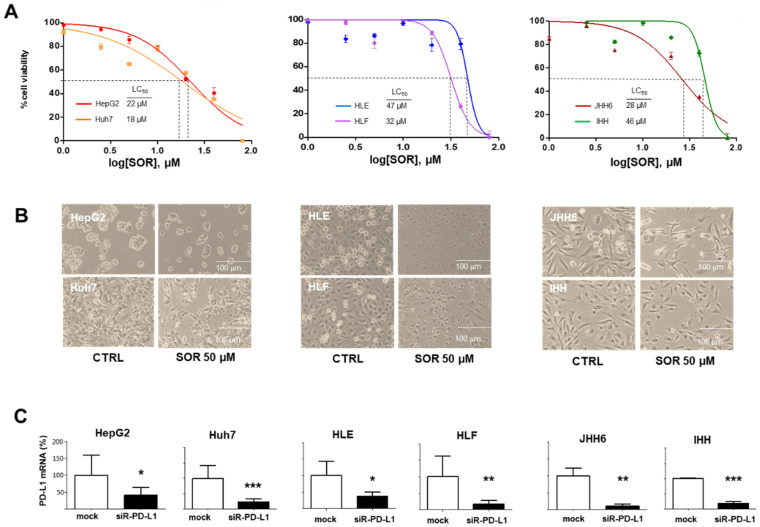
The Sorafenib and PD-L1 mRNA silencing treatments in in vitro models. (**A**) Cell viability upon 24 h treatment with 1 µM to 80 µM of SOR. Dashed lines show the value of LC_50_. (**B**) Cell morphology after 24 h treatment of 50 µM of SOR. (**C**) Down-regulation of PD-L1 mRNA expression after 48 h of 20 nM PD-L1 silencing. Graphs presented as mean ± SD calculated from at least three independent experiments. Statistical analysis: * *p* < 0.05, ** *p* < 0.01, *** *p* < 0.001 using Student’s *t*-test (vs. mock) in each cell line. SOR: Sorafenib, siR-PD-L1: PD-L1 silencing.

For the SOR, the following LC_50_ values shown in Figure 3A were calculated after 24 h of exposure of the cells to the drug. Cells belonging to the S2/progenitor subtype appear to be more sensitive to SOR as compared to cells belonging to S1/TGFβ-Wnt subtype. Noticeable morphological changes were observed in HLE, HLF, and JHH6 cells after treatment with 50 µM SOR (Figure 3B).

PD-L1 silencing and gene knockdown by siRNA resulted in a decrease of mRNA expression in all cell populations after 48 h of exposure to 20 nM of siR-PD-L1. Following RNA silencing, the extent of PD-L1 mRNA reduction was 70% and 64% for Huh7 and HepG2, respectively (*p* < 0.05). Higher extents of down-regulation were noticed in the S1/TGFβ-Wnt subtype cells, for 70%, 82%, and 91% for HLE, HLF, and JHH6, respectively (*p* < 0.05). PD-L1 down-regulation was also noticed for IHH cells for around 80% (*p* < 0.001) (Figure 3C).

### 3.4. Effect of Treatments on the Dysregulations of Targets

From the results of the cytotoxicity (5-AZA and SOR) and silencing experiments (siR-PD-L1), we further evaluated the dysregulations of the 16 proto-oncogene targets in Table 2 in the different cell populations. For the concentration of the treatments, concentrations of 5 µM [22] and 50 µM were selected for 5-AZA and SOR, respectively. For the silencing, the treatment with 20 nM of siRPD-L1 was able to significantly reduce PD-L1 mRNA expression in all cell lines investigated. We further evaluated the effect of these treatments on the target proto-oncogenes.

Among the three treatment modalities, 5 µM of 5-AZA did not show significant down-regulation effects on proto-oncogene targets on the different cells, except for FGR and PLZF. For SOR treatment, there were down-regulation effects in proto-oncogenes FGR, PLZF, and FOS. Interestingly, we also observed that the 50 µM SOR treatment down-regulated proto-oncogene mRNA expression mostly in the cells belonging to the S2/progenitor subtype. 

**Figure 4:**
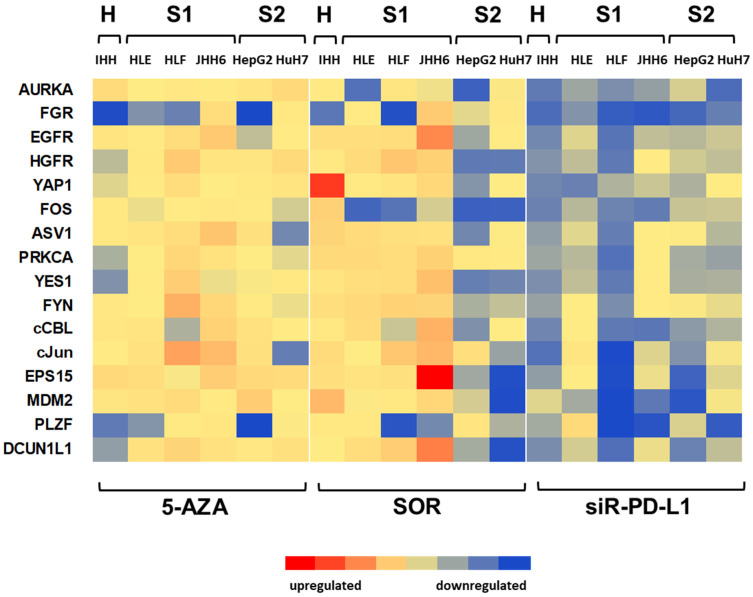
Dysregulation of targets mRNA expressions in liver cancer cell lines after various treatments. Heat map indicates the up-regulation and down-regulation of the markers after treatment with 5 µM 5-AZA, 50 µM SOR and 20 nM siR-PD-L1.

Notably, for the immune-targeting treatment results, using 20 nM siR-PD-L1 showed effective down-regulation in almost all proto-oncogenes in all cell lines evaluated. Figure 4 shows a representative heat map indicating the dysregulated mRNA expression of the proto-oncogenes. Exact values of the mean relative mRNA expression with corresponding statistical significance are shown in Supplemental Data. 

### 3.5. Dysregulation Effects of Various Treatments on FGR Protein Expression

For this initial analysis, we reported the effect of three treatment modalities on the expressions of FGR/Src, as a potential target. This molecule was down-regulated in almost all cell lines investigated after treatment with 5-AZA, SOR, and siR-PD-L1 (Figure 4 and Figure S2 of Supplemental Data). 

FGR mRNA was down-regulated in at least three cell lines following all treatments. 5-AZA was able to reduce FGR mRNA expression, ranging between 52% and 99% (*p* < 0.05) in four cell lines. SOR reduced its expression, ranging between 10% and 94% in three cell lines. Notably, sir-PD-L1-treated cells showed a significant reduction of FGR expression in all six cells lines, ranging between 51% and 89% (*p* < 0.001 for IHH, HLF, JHH6, and HepG2; *p* < 0.05 for HLE and Huh7). 

**Figure 5:**
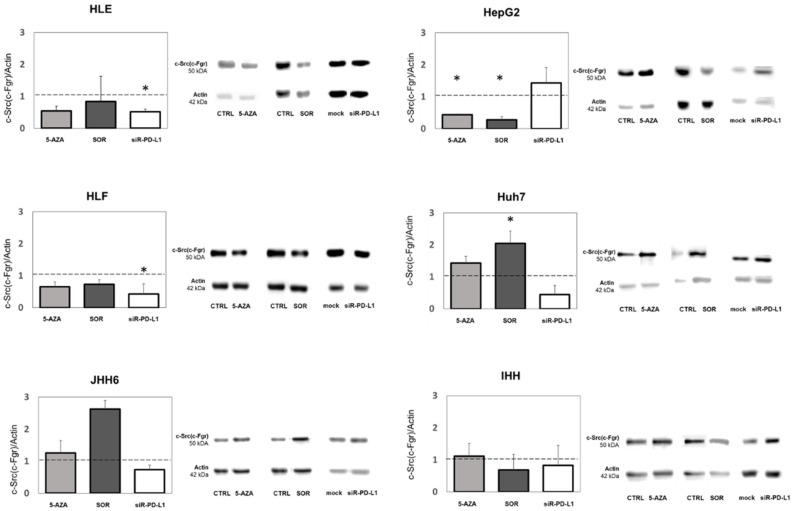
c-Src (c-Fgr) protein expression and its representative immunoblots among different HCC cell lines after treatment with 5-AZA, SOR and siR-PD-L1. Statistical analysis: * *p* < 0.05, Student’s *t*-test against CTRL or mock (=1.00; shown as dashed line). Actin (42 kDa) was used as a housekeeping protein against the c-Src (c-Fgr) (50 kDa).

The FGR mRNA expressions were then confirmed by Western Blot analysis. As shown in Figure 5, congruent results were noticed for both mRNA and protein expressions of Src family (including FGR). The antibody used was able to detect the protein expression of c-Src, c-Fgr, and other members of the Src family (50 kDa). With the exception of PD-L1-silenced HepG2 cells, the three treatment modalities reduced the c-Src protein expressions in almost all cell lines investigated.

## 4. Discussion

Treatment options for patients with advanced HCC are limited. At present, the available cancer therapies still offer modest effects on cancer treatment and patient survival. Besides a rather low efficacy, patients may also develop drug resistance. Furthermore, HCC is widely known as a vast heterogeneous tumor. Innate cellular heterogeneity of HCC largely contributes to the success of treatments. Moreover, it had been reported that intratumoral cellular and genetic differences exist from a slice of neoplastic tissue which in turn can influence the sensitivity to treatments [40].

Various attempts have been made to categorize HCC heterogenous tumors and classify them into groups that share common cellular and molecular profiles. Works of Hoshida et al., looked into clinical parameters such as tumor size, the extent of cellular differentiation, and serum α-fetoprotein levels and were able to suggest a robust subclassification of HCC. Their analysis of the signatures proposed three subclasses: (1) S1, marked by aberrant activation of the WNT signaling pathway; (2) S2, reflected by significant EpCAM positivity and also MYC and AKT activation; (3) S3, tumors classified by hepatocyte differentiation [15]. In parallel, Boyault et al., investigated transcriptome–genotype–phenotype profiles of HCC tumors and proposed a classification consisting of six subgroups (G1 to G6) based on their shared clinical and genetic profiles [16]. With these existing subclasses, Caruso et al.’s work evaluated liver cancer in vitro models to understand the diversity of HCC tumors and concluded that the experimental in vitro models could be reliable and viable tools to approach challenges in HCC biomarker discovery and drug response [3]. 

Utilizing these sets of information on HCC -omics heterogeneity, we carried out a strategy to identify potential putative markers for HCC treatment. Focusing our interest on cancer-promoting genes that are shared by the subclasses and subgroups, we evaluated, at the transcriptome level, 16 potential targets and their responses to three different treatment modalities on five different HCC cells.

Our study’s data confirmed the differences between subtypes of HCC, as shown from the profile of cancer stemness markers. From baseline mRNA expression analysis of the proto-oncogene targets on the different cell lines, our results showed that the S2/progenitor subtype displays more upregulated proto-oncogenes compared to S1/TGFβ-Wnt. This stratifies the existing differences between the two subtypes. 

Upon further analysis, more prominent up-regulations in proto-oncogenes were noted, such as, ASV, AURKA, and MDM2 in HCC cells compared to immortalized hepatocytes (Figure 2). It should be noticed that the activation, mutation, or overexpression of these genes had been reported to be involved in hepatocarcinogenesis [41,42,43]. We also observed proto-oncogenes that were down-regulated in the HCC cells, such as PLZF, YAP1, and FGR. Several publications had reported decreased expression of PLZF in HCC patients [29,44]. 

We then evaluated the above targets in in vitro experimental models using three treatment modalities. For epigenetic therapy using 5-AZA, the significant down-regulations were only noticed for FGR in three HCC cell lines. Moreover, down-regulation of PLZF was noticed in three cell lines, IHH, HLE and HepG2, after 5-AZA treatment, with a significant reduction only in the HepG2 cell line. Previously, it was reported that there was no association between promoter DNA methylation and PLZF gene expression in liver cancer [29]. However, in contrast in pancreatic cancer, the down-regulation of PLZF was associated with promoter DNA methylation of PLZF [45]. Since we showed the effect of DNA methylation inhibition on the gene expression of PLZF, our data might indicate an association between DNMT1 and PLZF, at least in several HCC cell lines. However, PLZF regulation might be influenced by other transcriptional silencing mechanisms, not only DNA methylation. More focused studies could be explored to understand promoter methylation of target proto-oncogenes to HCC. 

Regarding SOR treatment, our study showed significant proto-oncogene down-regulations, mostly noticed in cells belonging to the S2/progenitor subtype HepG2 and Huh7. This could suggest that the response to Sorafenib could be cellular/molecular subtype-directed. Particular molecular predictors, such as EpCAM and tuberous sclerosis complex-2 (TSC2), present in specific HCC subtypes, dictate the response to Sorafenib [46]. We had previously reviewed that cellular response to Sorafenib was affected by various factors such as genetic variants and differences in dysregulated molecules in tumor cells, eventually contributing to chemoresistance [4].

Immunotherapy is another targeted therapy that we evaluated in this study. In clinical practice, combination between Atezolizumab, an anti-PD-L1, and Cabonzantinib (anti-VEGFR) had shown potential as first-line treatment [12]. PD-L1, expressed primarily in cancer cells, was related to HCC prognosis [47,48]. In this study, we directly targeted the PD-L1 gene in cancer cells by silencing, which significantly reduced PD-L1 expression. In parallel, PD-L1 decrease was accompanied by the down-regulation of almost all investigated targets across all hepatic cells including for both HCC cell subtypes. This demonstrated an effective advantage of immune checkpoint (such as PD-L1) regulation compared to SOR or 5-AZA in terms of down-regulating cancer-promoting genes, at least in our datasets. Our data showed that this type of immune targeting was not dependent on cellular and molecular subtypes—which can be further utilized to overcome cancer heterogeneity.

To confirm the transcriptomic data, the protein expression of FGR/Src was measured by Western blot. The overexpression of FGR was previously reported to be significantly associated with poor prognosis in HCC [49]. As shown in Figure 5, the results of mRNA expression were in line with protein expression among all treatments and across various cell lines. Our GEPIA analysis (Figure S3 of Supplemental Data) showed that even though FGR expression was significantly down-regulated in HCC compared to normal tissues, low FGR expression might indicate a better survival of the patients [21], also supported by our data (Figure 2). 

The FGR data presented in this study is an initial data showing the relevance of the techniques to discover the potential target(s) in heterogeneous HCC cells. Further analysis of other significant targets (e.g., ASV, AURKA, MDM2, PLZF, FOS, etc.) will be equally important to search for the most prominent markers that can influence the heterogeneity of HCC cells. 

## 5. Conclusions

This study strengthens the relevance of HCC cellular heterogeneity in response to therapies and the identification of the relevant proto-oncogenes useful as new targets. Here we demonstrated that immune-targeted therapy by gene silencing demonstrates a treatment advantage in overcoming cellular heterogeneity. 

## Figures and Tables

**Table 1:** List of primers.

Target	Sequence F (5′ → 3′)	Sequence R (5′ → 3′)	Ref.
*YAP1*	CAATAGCTCAGATCCTTTCCT	TAGTATCACCTGTATCCATCTC	[24]
*AURKA*	GAGAATTGTGCTACTTATACTG	GGTACTAGGAAGGTTATTGC	ts
*FGR*	GGCCCGGCCTGCAT	TTGATGGCCTGAGAGGAGAAG	[25]
*EGFR*	AGGCACGAGTAACAAGCTCAC	ATGAGGGACATAACCAGCCACC	[26]
*MET, HGFR*	GGGCACCGAAAGATAAACCTCT	GACATTCTGGATGGGTGTTTCC	[27]
*YES1*	ACAGCAAGACAAGGTGCAAA	GTAAACCGACCATACAGTGCAG	[28]
*PLZF, ZBTB16*	TCACATACAGGCGACCACC	CTTGAGGCTGAACTTCTTGC	[29]
*DCUN1D1*	CTGGAGGACACCAACATG	TTCACTAGATTGTGTGAAGATC	[30]
*ASV, SRC1*	CGCTGGCCGGTGGAGTG	CCAGCTTGCGGATCTTGTAGT	[31]
*PRKCA*	GTGGCAAAGGAGCAGAGAAC	TGTAAGATGGGGTGCACAAA	[32]
*MDM2*	TTATTAAAGTCTGTTGGTGCA	TGAAGGTTTCTCTTCCTGAAG	[33]
*FOS*	CCGGGGATAGCCTCTCTTAC	GTGGGAATGAAGTTGGCACT	[34]
*CBL*	TGCCAAAACTGCCACCTGGGG	GGGCTGCGGCCAAATTCCCT	[35]
*FYN*	GGACATGGCAGCACAGGTG	TTTGCTGATCGCAGATCTCTATG	[36]
*JUN*	AAGTAAGAGTGCGGGAGGCA3	GGGCATCGTCATAGAAGGTCG	[37]
*EPS15*	CCTGTTGCAGATTTCTCTG	TCATCTTGAAGATCCTGAAC	[38]
*ACTB*	CGCCGCCAGCTCACCATG	CACGATGGAGGGGAAGACGG	ts
*PD-L1*	AAAGTCAATGCCCCATACAA	ACATGTCAGTTCATGTTCAGAG	[39]

ts: this study.

**Table 2:** List of targets and their expression in LIHC.

UNIPROT ID	Protein Name	Gene	Gene Name	* Gene Expression
P46937	Transcriptional coactivator YAP1	YAP1	yes-associated protein 1	no difference
O14965	Aurora kinase A	AURKA	aurora kinase A	upregulated
P09769	Tyrosine-protein kinase Fgr	FGR	FGR proto-oncogene	down-regulated
P00533	Epidermal growth factor receptor	EGFR	epidermal growth factor receptor	no difference
P08581	Hepatocyte growth factor receptor	HGFR, MET	MET proto-oncogene, receptor tyrosine kinase	upregulated
P07947	Tyrosine-protein kinase Yes	YES1	YES proto-oncogene 1, Src family tyrosine kinase	upregulated
Q05516	Zinc finger and BTB domain containing 16	PLZF, ZBTB16	zinc finger and BTB domain containing 16	down-regulated
Q96GG9	DCN1-like protein 1	DCUN1D1	defective in cullin neddylation 1 domain containing 1	upregulated
P12931	Proto-oncogene tyrosine-protein kinase Src	ASV, SRC1	SRC proto-oncogene, non-receptor tyrosine kinase	upregulated
P17252	Protein kinase C alpha type	PRKCA	protein kinase C alpha	upregulated
Q00987	E3 ubiquitin-protein ligase Mdm2	MDM2	MDM2 proto-oncogene	upregulated
P01100	Protein c-Fos	FOS	Fos proto-oncogene, AP-1 transcription factor subunit	down-regulated
P22681	E3 ubiquitin-protein ligase CBL	CBL	Cbl proto-oncogene	upregulated
P06241	Tyrosine-protein kinase Fyn	FYN	FYN proto-oncogene, Src family tyrosine kinase	down-regulated
P05412	Transcription factor Jun	JUN	Jun proto-oncogene, AP-1 transcription factor subunit	upregulated
P42566	Epidermal growth factor receptor substrate 15	EPS15	epidermal growth factor receptor pathway substrate 15	upregulated

* Data from GEPIA—Gene Expression Profiling Interactive Analysis (GEPIA); TCGA Data LIHC = 369 vs. TCGA and GTex Data Normal = 160); LIHC—Liver hepatocellular carcinoma.

## Data Availability

Data are available upon reasonable request.

## Supplementary Materials

The following supporting information can be downloaded at: https://www.mdpi.com/2227-9059/11/2/342/s1, Figure S1. Protein-protein interaction (PPI) analysis of the different HCC subtypes and groups from published datasets. Figure S2. Mean mRNA expression data from at least three independent replicates showing the dysregulation of proto-oncogene upon treatment from the cell lines investigated (including result of statistical analysis). Figure S3. The relevance of FGR in clinical samples of LIHC (gene expression distribution (normal vs LIHC) and overall survival data from GEPIA web tool).
