# Supplementary material for: Network Analysis for the Discovery of Common Oncogenic Biomarkers in Liver Cancer Experimental Models
Source: Biomedicines. 2023 Jan 25;11(2):342. doi: 10.3390/biomedicines11020342 (PMC9953082; doi:10.3390/biomedicines11020342)
Supplement: Supplementary file 1 [file biomedicines-11-00342-s001.zip › biomedicines-2116246-supplementary.pdf]

Network analysis for the discovery of common oncogenic biomarkers in liver cancer experimental models

Loraine Kay D. Cabral[1,2,*], Pablo J. Giraudi[1], Gianluigi Giannelli[3], Francesco Dituri[3], Roberto Negro[3], Claudio Tiribelli[1] and Caecilia H. C. Sukowati[1,4]

[1] Fondazione Italiana Fegato ONLUS, AREA Science Park, Campus Basovizza, 34149 Trieste, Italy

[2] Doctoral School in Molecular Biomedicine, University of Trieste, 34127 Trieste, Italy

[3] National Institute of Gastroenterology "S. De Bellis" Research Hospital, 70013 Bari, Italy

[4] Eijkman Research Center for Molecular Biology, National Research and Innovation Agency of Indonesia (BRIN), B.J. Habibie Building, Jl. M.H. Thamrin No. 8, Jakarta Pusat 10340, Indonesia

* Correspondence: caecilia.sukowati@fegato.it

Supplemental Data

<u>Hoshida *et al.* datasets</u>

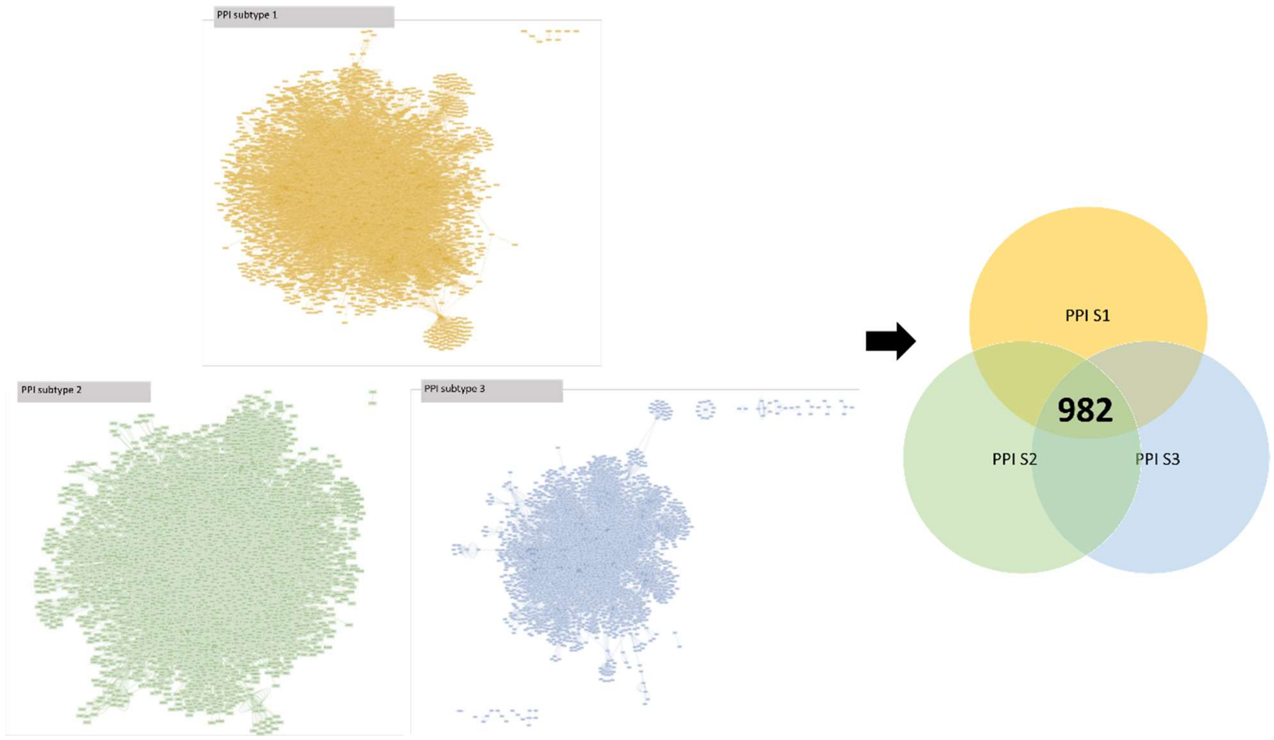

<u>Boyault *et al.* datasets</u>

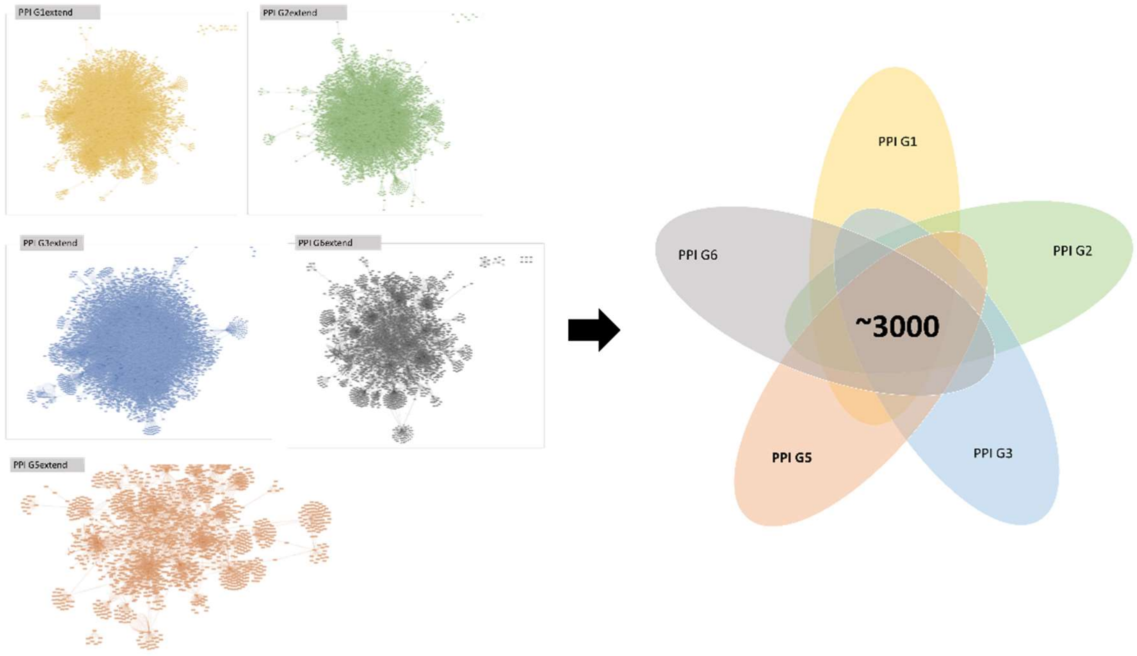

Figure S1. Protein-protein interaction (PPI) analysis of the different HCC subtypes and groups from published datasets.

5-AZA

	IHH (H)	HLE (S1)	HLF (S1)	JHH6 (S1)	HepG2 (S2)	HuH7 (S2)
AURKA	3.00	106	146	1.38	1.96	3.26 **
FGR	0.04	0.48 **	0.37 *	2.84	0.01 *	1.51
EGFR	1.99	140 **	2.74	5.14	0.77	1.18
HGFR	0.75	165	5.04	1.91	1.89	3.13
YAP1	0.91	1.35	2.80 *	1.08	1.55	2.00
FOS	1.58 *	0.97	1.29	1.45	1.30	0.85
ASV1	1.51	2.01	2.79	5.80 *	2.41 **	0.42
PRKCA	0.67	1.14	3.47	2.36 *	1.08	0.93
YES1	0.48	1.24	4.71	0.97	1.03	1.55
FYN	1.48	1.14	8.11	3.55 *	1.30	0.97
cCBL	1.68	1.90	0.68	4.09	2.27	1.21
cJun	1.28	2.23	9.81***	6.87 **	2.43	0.35 **
EPS15	3.32	2.84	1.03	4.57 **	3.28	3.06
MDM2	1.86	2.28 **	3.04	1.35	4.88 *	1.13
PLZF	0.33	0.49	1.40	1.76	0.01 **	1.04
DCUN1L1	0.56	2.33	3.99	2.18	1.47	2.45

SOR

	IHH (H)	HLE (S1)	HLF (S1)	JHH6 (S1)	HepG2 (S2)	HuH7 (S2)
AURKA	1.39	0.27 *	1.83	0.99	0.17 **	1.04
FGR	0.31	1.25	0.06 *	4.91 ***	0.93	1.44
EGFR	2.67	2.61	2.86 **	13.01 *	0.62	1.24
HGFR	1.59	3.06	5.56	4.28 *	0.32 *	0.33
YAP1	22.36	1.35	2.00	3.40	0.50	1.06
FOS	4.18	0.20 *	0.29 *	0.86	0.16 **	0.17
ASV1	3.99	3.09 *	2.41 *	2.16	0.41 *	1.05
PRKCA	3.18	3.30	2.80 **	4.28	1.58	1.37
YES1	1.96	2.60 *	2.76	6.26 *	0.36 *	0.40
FYN	2.63	3.32 *	4.08 **	4.03 **	0.67	0.78
cCBL	1.50	3.01	0.82	7.95	0.47	1.12
cJun	3.00	1.15	5.44	7.17 *	2.68	0.59
EPS15	2.57	2.09	2.43	29.21	0.62	0.05
MDM2	7.16	1.04	1.65	3.65 *	0.85	0.04
PLZF	1.61	1.48	0.09	0.42	1.91	0.68
DCUN1L1	1.11	2.87	4.73	14.08 **	0.64	0.06 *

siR-PD-L1

	IHH (H)	HLE (S1)	HLF (S1)	JHH6 (S1)	HepG2 (S2)	HuH7 (S2)
AURKA	0.33***	0.61	0.46 **	0.57 **	0.87	0.24***
FGR	0.24***	0.49 *	0.14***	0.11 ***	0.21***	0.36 *
EGFR	0.42***	0.91 *	0.29 *	0.77	0.73	0.82
HGFR	0.49 **	0.76	0.32 **	1.19	0.84	0.77
YAP1	0.41	0.37	0.69 **	0.81	0.68	1.13
FOS	0.38***	0.73***	0.39***	0.34	0.80	0.82
ASV1	0.56***	0.92	0.35***	1.13	1.05	0.72
PRKCA	0.61	0.73 **	0.27 **	1.06	0.65 **	0.58
YES1	0.46 **	0.77	0.33 *	1.21	0.66***	0.68
FYN	0.58 **	1.08	0.45 *	1.24	1.04	0.95
cCBL	0.40 *	1.17	0.32	0.30	0.53 *	0.70 *
cJun	0.28 **	1.82	0.02***	0.90	0.48	1.02
EPS15	0.56	1.07	0.03***	0.97	0.18	0.91 **
MDM2	0.92	0.64	0.02***	0.32 *	0.09	1.02 **
PLZF	0.64	3.20***	0.02***	0.08***	0.88 **	0.13
DCUN1L1	0.45	0.85	0.26 *	0.97	0.38	0.77 **

upregulated — downregulated

** p=<0.05*
***p=<0.01*
****p=<0.001*

Figure S2. Mean mRNA expression data from at least three independent replicates showing the dysregulation of proto-oncogene upon treatment from the cell lines investigated. Statistical analysis to evaluate the difference between means vs control/untreated cells is indicated in the corresponding p-value (*p < 0.05, **p < 0.01, ***p < 0.001).

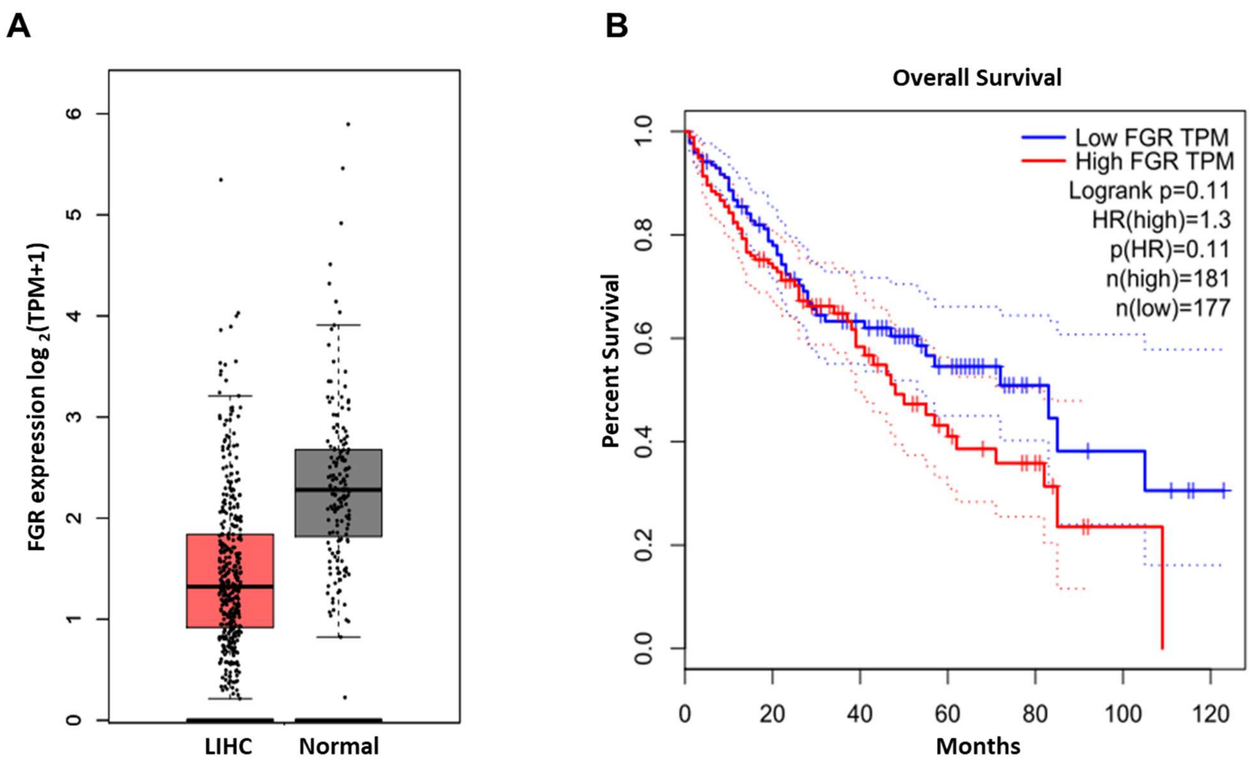

Figure S3. The relevance of FGR in clinical samples of LIHC. (**A**)The distribution of FGR in tumoral samples compared to non-tumoral tissues. (**B)** The association of FGR expression in the overall survival of patients. Datasets were taken from TCGA and GTEx databases and visualized by GEPIA online tool. LIHC - Liver hepatocellular carcinoma.
